# Early menopause, association with tobacco smoking, coffee consumption and other lifestyle factors: a cross-sectional study

**DOI:** 10.1186/1471-2458-7-149

**Published:** 2007-07-07

**Authors:** Thea F Mikkelsen, Sidsel Graff-Iversen, Johanne Sundby, Espen Bjertness

**Affiliations:** 1 Institute of General Practice and Community Medicine, Faculty of Medicine, University of Oslo, Norway; 2 Norwegian Institute of Public Health, Oslo, Norway

## Abstract

**Background:**

Early onset of menopause is a risk factor for several health problems. The objective was primarily to investigate the association between early menopause and current, past active and passive smoking. A second aim was to investigate the association between coffee and alcohol consumption and early menopause.

**Methods:**

The present population-based cross-sectional study included a sub-sample of 2123 postmenopausal women born in 1940–41 who participated in the Oslo Health Study. Early menopause was defined as menopause occurring at an age of less than 45 years. We applied logistic regression analyses (crude and adjusted odds ratio (OR)) to examine the association between early menopause and selected lifestyle factors.

**Results:**

Current smoking was significantly associated with early menopause (adj. OR, 1.59; 95% CI, 1.11–2.28). Stopping smoking more than 10 years before menopause considerably reduced the risk of early menopause (adj. OR, 0.13; 95% CI, 0.05–0.33). Total exposure to smoking (the product of number of cigarettes per day and time as a smoker) was positively related to early menopause and, at the highest doses, nearly doubled the odds (adj. OR, 1.93; 95% CI, 1.12–3.30). These data suggest a possible dose-response relationship between total exposure to smoking and early menopause, but no dose-response relationship was detected for the other variables examined. We found no significant association of coffee or alcohol consumption with early menopause. Of the lifestyle factors tested, high educational level (adj. OR, 0.50; 95% CI, 0.34–0.72) and high social participation (adj. OR, 0.60, 95% CI, 0.39–0.98) were negatively associated with early menopause.

**Conclusion:**

This cross-sectional study shows an association between current smoking and early menopause. The data also suggest that the earlier a woman stops smoking the more protected she is from early menopause. Early menopause was not significantly associated with passive smoking, or alcohol or coffee consumption.

## Background

Early onset of menopause is a risk factor of osteoporosis [1] and death from ischemic heart disease [2], although it seems to protect against breast cancer [3,4]. These effects may result from an earlier decrease in the amount of female sex hormones [5,6]. Today, women tend to want children later and the age of giving birth is getting closer to the age of menopause. In Northern Europe, the mean age of the firstborn child is 27.1 years. In Oslo it is as high as 28.9 years, and 2.3% of Norwegian women have children after the age of 40 [7]. Early menopause is becoming an important public health issue as a risk factor for fertility problems.

During the last two decades, several risk factors for early menopause have been investigated. The results are inconsistent, but the trend suggests that nulliparous women have an earlier onset of menopause than do parous women [8,9]. A high educational level [10,11], being overweight [8-10], a moderate intake of alcohol [10,12] and a higher intake of coffee [9] seems to protect against early menopause. Current smoking around the onset of menopause is an established risk factor for early menopause [10,13-16], whereas previous smoking seems to matter less [8,11,13,14,16]. Some studies have reported a dose-response effect of smoking on early menopause [8,16,17], although other studies show no such effect [11,13]. Among the few published studies of passive smoking and ovarian function, Everson et al. found that both passive and active smoking are associated with early menopause [18], whereas Cooper et al. [13] and Cramer et al. [17] reported an association with active smoking, but not with passive smoking.

Detailed information was collected in 2000–01 from 59–60-year-old women from Oslo about their past and present smoking habits, coffee and alcohol use, educational level and other factors relevant to health. The prevalence of smoking, both active and passive, is high in this cohort. The objective of this study is to investigate the association between early menopause and active and passive smoking including a possible dose-response relationship with active smoking and early menopause. One further main aim of this study is to investigate the association between early menopause and coffee and alcohol consumption.

## Methods

The data for this analysis were obtained from The Oslo Health Study conducted in the city of Oslo from May 2000 to September 2001. The sampling and data collection have been described in detail elsewhere [19]. All inhabitants registered with an address in Oslo and born in the years 1970, 1960, 1955, 1940–41 and 1924–25 were invited to participate in the study, which included a questionnaire and a simple clinical examination. The Norwegian Data Inspectorate has approved The Oslo Health Study, the Regional Committee for Medical Research Ethics has evaluated it, and it has been conducted in full accordance with the World Medical Association Declaration of Helsinki.

Data from women born in 1940–41 were analysed for the present study. Of 4116 invited women in this group, 2357 participated (57.3%), all with full written consent. We excluded 159 women because of missing relevant information, most often on menopausal age and in some cases on the age of menarche. Another 75 were excluded because they reported a menopausal age of less than 40 years, as it is likely that a large percentage had undergone surgical menopause. The total number of participants was 2123, 51.6% of the total population in the age group.

The participants were asked whether they were current smokers, former smokers or never smokers. The current and former smokers reported the number of cigarettes they smoked per day and at which age they started smoking on a daily basis. The former smokers also reported the number of years since they stopped smoking. With the help of the latter variable and the self-reported age of menopause, we estimated their smoking status at the age of menopause and during the years before. Total smoking exposure was estimated by multiplying the number of cigarettes smoked daily by 365 and then by the number of years of active smoking. Participants were divided into three categories of active exposure to tobacco smoking: low (365–64,240 cigarettes), medium (64,241–158,775 cigarettes) and high (158,776–657,000 cigarettes) exposure.

Passive smoking was recorded by asking participants: (1) how many hours a day was currently spent in a room where people smoke, (2) whether they had a smoking mother or father while growing up and (3) whether after the age of 20 they had lived in a household with anybody who smoked. Never-smokers who regularly spent time in the same room as smokers or answered yes to one of these two last questions, were defined as passive smokers.

Participants were divided into three categories based on the reported age when the menses had stopped: early menopause (40–44 years), median menopause (45–54 years) and late menopause (>54 years). The participants were not asked about the cause of menopause – that is, if the menopause was natural or surgical or due to other medical treatment.

The participants' educational level was divided into four levels: primary school, junior secondary school, senior secondary school or university education. Marital status was categorized as: married, unmarried, widowed, or divorced or separated.

Social participation was measured by the number of formal or informal organizations or clubs they belonged to. Low participation was defined as no membership, normal participation as membership in 1–3 organizations or clubs, and high participation as involvement in >3 organizations or clubs.

Body weight (to the nearest 100 g) and height (to the nearest mm) was measured with an electronic height and weight scale. Body mass index (BMI) was obtained by dividing body weight in kg by height in m^2^. According to WHO standards, BMI < 18.5 was considered underweight, 18.5–24.9 normal weight, 25–29.9 overweight and ≥30 obese [20].

The participants were asked how many hours a week they performed physical activity that made them sweat or out of breath. Based on their answer, they were divided into four groups (no such physical activity, ≤ 1 hour, 1–2 hours and ≥ 3 hours).

Data were analysed using SPSS 12.0 for Windows. We applied logistic regression analysis (crude and adjusted OR) and set the level of significance as p < 0.05 and 95% CI. The associations were adjusted for potential confounders, including education and selected lifestyle factors. The potential interaction of education with smoking was investigated by including the product term of these factors in the regression analysis, which was non-significant.

## Results

In this cohort, the mean age of menopause was 50.1 years and the median age was 50 years. The range was 40–61 years. The 25th percentile was 48 years and the 75th percentile was 53 years. In all, 9.6% of the women had early menopause.

In this group active and passive smoking was frequent. 24.2% were current smokers, 28.7% were former smokers and 35.2% were defined as passive smokers.

Table 1 presents the crude associations between early menopause and selected risk factors. Women with a higher educational level were less likely to have entered menopause at an early stage (OR, 0.37; 95% CI, 0.23–0.60). At the same time the women with the highest level of education are the ones the least exposed to smoking. Among the ones with more than 12 years of education, the percentage of never smokers is 54.0% and the percentage of current smokers is 16.9%. These same percentages among the women with less than 8 years of education are respectively 40.8% and 33.1%. Being widowed as compared to being married almost doubled the odds for early menopause (OR, 1.89; 95% CI, 1.18–3.02), and a self-reported worse general health showed a similar association (OR, 1.83; 95% CI, 1.36–2.47).

**Table 1:** Association between early menopause (40–44 years) and selected variables

Variables	% with early menopause and total number that answered		Crude OR	95% CI	
	%	N		Lower	Upper

**Marital status**					
Married	8.5	1186	Ref.		
Unmarried	7.4	204	0.79	0.45	1.39
Widowed	15.2	171	1.89	1.18	3.02
Divorced or separated	10.9	558	1.32	0.94	1.86
**Parity**					
0	9.9	304	Ref.		
1–3	9.8	1618	1.03	0.68	1.56
>3	7.1	168	0.72	0.36	1.45
**Age at menarche**					
12–15	9.1	1773	Ref.		
<12	12.5	168	1.44	0.88	2.35
>15	11.5	182	1.36	0.83	2.22
**Years of education**					
<8	18.4	163	Ref.		
8–10	10.3	630	0.51	0.32	0.82
11–12	9.6	426	0.50	0.30	0.83
>12	7.3	874	0.37	0.23	0.60
**Social participation**					
Low	11.3	964	Ref.		
Medium	8.3	931	0.72	0.53	0.98
High	5.4	148	0.49	0.23	1.04
**Self reported health**					
Good	7.8	1354	Ref.		
Bad	12.9	728	1.83	1.36	2.47
**Body mass index**					
18.5–24.9	9.3	965	Ref.		
<18.5	16.8	18	1.95	0.55	6.98
25.0–29.9	9.8	724	1.12	0.81	1.56
≥30	9.5	367	1.13	0.74	1.70
**Physical activity (hours per week)**					
0	10.7	844	Ref.		
<1	8.7	412	1.02	0.59	1.75
1–2	7.6	406	0.79	0.43	1.44
≥3	10.8	167	0.70	0.38	1.30

Other variables, such as age at menarche, parity, BMI or physical activity were not significantly correlated with age of menopause. Adjustment in relevant analyses for marital status, educational level, social participation, self reported health and coffee consumption changed the point estimates by 0.1–0.2, but the significance levels were not much affected. Thus only the crude ORs are presented in table 1.

Table 2 presents data of the association between menopause and stimulants (smoking, coffee and alcohol). Current active smoking showed an association with early menopause when adjusted for education (adj. OR, 1.59; 95% CI, 1.11–2.28). This association did not change after further adjustment for the intake of coffee and alcohol, but when adjusting in addition for marital status, educational level, social participation, self reported health and coffee consumption the adj. OR is 1.33 and 95% CI 0.85–2.08.

**Table 2:** Association between early menopause and smoking and intake of coffee and alcohol

Variable	% with early menopause and total number that answered		Crude OR	95% CI		Adj. OR ^a^	95% C.I.		Adj. OR ^b^	95% CI	
	%	N		Lower	Upper		Lower	Upper		Lower	Upper

**Smoking**											
Never	7.8	963	Ref.			Ref.			Ref.		
Earlier	9.2	609	1.18	0.82	1.71	1.16	0.80	1.68	1.27	0.86	1.88
Current	13.4	514	1.71	1.21	2.42	1.59	1.11	2.28	1.66	1.12	2.45
**Coffee (cups per day)**											
0	7.9	280	Ref.			Ref.			Ref.		
1–4	8.8	1431	1.15	0.72	1.85	1.14	0.70	1.86	1.09	0.66	1.79
>4	13.6	338	1.83	1.07	3.14	1.64	0.94	2.88	1.31	0.73	2.37
**Alcohol consumption**											
None in last year	12.2	172	Ref.			Ref.			Ref.		
<1 time per week	10.5	849	0.80	0.48	1.34	0.93	0.55	1.59	0.83	0.47	1.47
At least once per week	8.4	1083	0.62	0.37	1.04	0.78	0.46	1.34	0.66	0.37	1.18

^a ^Adjusted for educational level

^b ^Adjusted for the two other stimulants and educational level

To drink more than four cups of coffee a day showed an association with early menopause (OR, 1.83; 95% CI, 1.07–3.14), but this association disappeared when adjusting for the other variables shown to be related with the end point (adj. OR 1.58; 95% CI 0.88–2.83, data not shown) or the two other stimulants (adj. OR 1.31; 95% CI 0.73–2.37) (table 2). A part from being borderline significantly negatively associated with menopause in the crude analyses, alcohol consumption showed no association with the onset of menopause (table 2).

Table 3 shows the associations between active smoking and early menopause. The number of cigarettes smoked daily (adj. OR, 1.48; 95% CI, 1.03–2.13) was positively associated with early menopause and even shows a dose-response relationship. A test for linear trend turned out positive (p-value = 0.04). The number of years between smoking cessation and the age of menopause was also significantly associated with age of menopause (Table 3); women who had stopped smoking more than 10 years before menopause had a lower risk of early menopause than did those still smoking at the start of menopause (adj. OR, 0.13; 95% CI, 0.05–0.33). Total exposure to smoking analysed among current and former smokers, was positively related to the age of menopause, and women at the highest doses had nearly double the odds of early menopause (adj. OR, 1.79; 95% CI, 1.06–3.02).

**Table 3:** Association between early menopause and exposure to tobacco smoke

Variable	% with early menopause and total number that answered		Crude OR	95% CI		Adj. OR ^a^	95% CI	
	%	N		Lower	Upper		Lower	Upper

**Number of cigarettes smoked daily**								
0	7.8	966	Ref.			Ref.		
1–10	9.7	610	1.24	0.86	1.78	1.17	0.81	1.68
>10	12.1	514	1.54	1.08	2.21	1.48	1.03	2.13
**Total smoking exposure ^b^**								
Low exposure	7.2	334	Ref.			Ref.		
Medium exposure	12.1	354	1.73	1.02	2.94	1.70	1.00	2.89
High exposure	13.2	364	1.82	1.08	3.06	1.79	1.06	3.02
**Age when started daily smoking **(current smokers. N = 514)								
<20	14.9	235	Ref.			Ref.		
20–24	14.0	164	0.97	0.55	1.73	0.95	0.52	1.73
>24	10.5	105	0.72	0.35	1.50	0.73	0.35	1.53
**Age when started daily smoking **(former smokers. N = 609)								
<20	10.5	286	Ref.			Ref.		
20–24	8.1	197	0.71	0.37	1.35	0.68	0.35	1.32
>24	6.4	110	0.58	0.25	1.37	0.59	0.25	1.40
**Number of years before the age of menopause one stopped smoking **(former smokers. N = 609)								
Smoking at the time of menopause	16.9	195	Ref			Ref.		
1–10	11.9	135	0.74	0.39	1.42	0.68	0.35	1.32
>10	2.3	256	0.14	0.06	0.34	0.13	0.05	0.33

a) Adjusted for educational level

b) Number of cigarettes per year × years smoked

The ORs in Table 3 were changed by 0.1–0.4 by inclusion of marital status, educational level, social participation, self reported health and coffee consumption as covariates. The most pronounced change was seen for high total exposure to smoking (adj. OR 1.47 (95-% CI 0.77–2.79). The result concerning smoking cessation was practically not changed (adj. OR 0.13; 95-% CI 0.05–0.36 by cessation of smoking >10 years prior to menopause).

Separate analysis of those smoking at the age of menopause showed an inverse association between the number of years of active smoking and early onset of menopause (OR, 0.89; 95% CI, 0.86–0.92, adjusted for education, data not shown). Age of starting to smoke on a daily basis was not associated with early menopause.

Passive smoking recorded among non-smokers was not significantly associated with early menopause except for a borderline significant association of parents' smoking when growing up (Table 4). The inclusion of other variables presented in Table 1 as covariates did not change the significance levels.

**Table 4:** Association between early menopause and passive exposure to tobacco smoke among never smokers

Variable	% with early menopause and total number that answered		Crude OR	95% CI		Adj. OR ^a^	95% CI	
	%	N		Lower	Upper		Lower	Upper

**Hours present in a room where people are smoking**								
0	7.6	802	Ref.			Ref.		
1–6	9.2	76	1.28	0.56	2.94	1.04	0.44	2.44
>6	6.3	16	1.12	0.14	8.99	0.99	0.12	8.12
**Smoking mother or father while growing up**								
No	9.0	378	Ref			Ref.		
Yes	6.1	580	1.50	0.90	2.50	1.56	0.92	2.64
**Living with smokers after the age of 20**								
No	7.7	533	Ref.			Ref.		
Yes	8.2	417	1.07	0.66	1.73	0.94	0.58	1.54

^a ^Adjusted for educational level

## Discussion

Our study of postmenopausal women is population based with a high proportion of smoking participants. Our main finding was that current active smoking is related to early menopause, and that smoking cessation prior to menopausal age seems to protect against early menopause.

The most important limitation of our study is that the women were 59–61 years when they participated in the survey and were asked about health and related habits in their *current *situation. Many of the factors examined at that age, including BMI, coffee and alcohol consumption and marital status, may not reflect the status of these factors in the years before and around menopause. The reports on coffee and alcohol consumption or current smoking at the time of the survey were, however, probably representative for the status around menopausal age, and any misclassification of these variables would most probably be non-differential. In the Tromsø Heart Study the relative validity of the question on coffee consumption per day was compared with a dietary history survey two years later and the answers agreed well for coffee as well as other food items used every day in easily recorded unites [21]. Also, the reproducibility of the Tromsø survey questionnaire was studied by comparing it with a new questionnaire assessment one year later, and the concordance of coffee consumption was 67% for exact agreement and 99% for agreement within one category (Rho = 0.65). In the same study the concordance of alcohol consumption (beer, wine and spirit were recorded separately) was 66–76% for exact agreement and 96–100% for agreement within one category [22].

Previous studies have reported that high body weight is associated with later menopause [8,9]. However, we found no association between BMI and menopause, possibly because of changes in BMI between the onset of menopause and the survey.  It has been reported that the menopause increases the body weight [23] whereas other authors state that menopause is not affecting the BMI, merely the body composition and fat distribution [24]. Earlier smoking habits were reported and if misclassification occurred, it was probably not related to menopausal age. Any effect of these limitations would be to reduce the strength of the associations.

A potential limitation is recalling the exact age at which menopause occurred. Studies have shown that the reported age of menopause may be inaccurate [25,26], although other studies have concluded that the event is so important in a woman's life that she will remember it clearly [27,28]. However, even if the reporting is inaccurate, there is no systematic age misclassification [25] and any recall problem should reduce the strength of the associations. The use of hormone-replacement therapy was low in Norway until 1990, and then rose gradually to 16% of women aged 45–69 years in 1994 [29]. If started before any menopausal symptoms, hormone use may conceal the cessation of ovarian function and may lead a woman to not correctly estimate her age at menopause. However, this possible source of bias is of little relevance for the subgroup of women with menopause at the age of 40–44 years, and the low rates of hormone treatment make it unlikely that the menopausal age was misclassified in this cohort.

The response rate was 57.3% and selection bias is expected with this rate. However, it was conducted a study of self-selection based on all invited to The Oslo Health Study linking the socio-demographic data from public registers in Statistics Norway and data from The Oslo Health Study [19]. The response rate was positively associated with educational attainment, total income, married status and a western country of birth, and negatively associated with receiving disability benefit. Self-selection according to socio-demographic variables had little impact on the prevalence estimates of self-rated health, smoking and BMI [19]. There is no evidence or indication of connection between survey participation and menopausal age, so that self-selection for participation had probably little or no impact on the associations we found. Moreover, it has been shown that less educated persons were less likely to participate in the Oslo Health Study than were higher educated, but further that this had probably little impact even on prevalence data for risk factors such as smoking [19]

There may also be a certain survival bias. It can be speculated that individuals who are genetically the most vulnerable for smoking-related disease will be overrepresented among those dying before the age of 60. However, this most likely concerns few individuals and should reduce the association.

We lacked information about whether the menopause was natural or surgical. We excluded women who reported reaching menopause before the age of 40 years because it is likely that their menopause resulted from surgery, but it is also likely that some women in the sample underwent surgery after the age of 40 years. However, the rate of hysterectomy and oophorectomy is low in Norway, by a factor three to four, compared with the United States [30]. A large cohort study in Norway in 1995–97 reported that 10% of menopausal women aged 50–59 years had undergone a simple hysterectomy [31], and we consider 10% as a maximum estimate of the prevalence of women that might have been misclassified into the early menopause group in our study.

We have adjusted for most of the variables thought to influence age at menopause. It is possible that adjusting for variables such as stress, menstrual cycle patterns and family history of age at menopause could be relevant, but we had no information on these factors.

The results concerning current active smoking, number of cigarettes smoked daily and total smoking exposure differed by whether we adjusted for education alone or for all variables associated with menopausal age. The latter model may be an over-adjustment as not all of these variables have been shown to really influence menopausal age. In light of the present evidence one can argue that the adjustment for education only gives the least chance for a false result.

Similar to Cooper et al. [13], we did not find that passive exposure to smoking was associated with early menopause. This contrasts with the study by Everson et al[18] and with Cooper's earlier findings [32]. We included never-smokers only in the analysis of passive smoking. Cooper et al. also excluded current smokers in the analyses of passive smoking in the later paper [13], whereas former smokers were included in the earlier paper [32]. This may partially explain the different results. Everson et al. included only never-smokers, but still found an association between passive smoking and early menopause.

We found no association between early menopause and alcohol or coffee consumption. However, the crude OR showed that coffee consumption is associated with an early onset of menopause, which is consistent with Nagata et al.'s findings [9]. Nagata et al. adjusted for a number of factors, but not educational level or smoking, which we found to be important confounders of coffee consumption. When we adjusted for these factors, coffee was no longer associated with the onset of menopause, which is consistent with the findings of other studies [17,33].

Other studies have reported that a moderate intake of alcohol is associated with later menopause [10,12,33]. However, only one of these studies adjusted for both smoking and education. Our analysis showed that education is an important confounder of the association between alcohol consumption and early menopause. If adjusted only for smoking and coffee consumption, alcohol was associated with early menopause (adj. OR, 0.56; 95% CI, 0.32–0.98, data not shown), but this association was no longer significant when adjusted for educational level.

## Conclusion

Our study showed an association between current smoking and early onset of menopause, and that the earlier a woman stops smoking, the more protection she derives with respect to an early onset of menopause. Passive smoking and alcohol or coffee consumption were not significantly associated with early onset of menopause.

## Competing interests

The author(s) declare that they have no competing interests.

## Authors' contributions

TFM, SGI, JS and EB participated in the design of the study and in writing the manuscript.

TFM performed the statistical analyses

All authors read and approved the final manuscript.

## Pre-publication history

The pre-publication history for this paper can be accessed here:


